# Preparation, Sensory, and Nutritional Evaluation of Extruded Functional Food Products Using Asparagus racemosus (Shatavari)

**DOI:** 10.7759/cureus.79593

**Published:** 2025-02-24

**Authors:** Aparna Srivastava, Rumana Ahmad, Arvind K Srivastava

**Affiliations:** 1 Department of Food and Nutrition, Era University, Lucknow, IND; 2 Department of Biochemistry, Era's Lucknow Medical College and Hospital, Lucknow, IND

**Keywords:** asparagus racemosus roots, extrusion cooking, nutritional analysis, semolina pasta, sensory evaluation, whole-wheat noodles, whole-wheat pasta

## Abstract

Introduction: Functional foods provide health benefits beyond basic nutrition, with medicinal plants like *Asparagus racemosus *(*A. racemosus*) (Shatavari) offering therapeutic potential. This study investigates the incorporation of *A. racemosus* root powder into noodles and pasta to create nutrient-rich functional foods.

Methods: *A. racemosus* roots were dried, powdered, and incorporated into noodles and pasta formulations at varying levels (0%, 5%, 10%, and 15%). Sensory evaluation for color, texture, taste, and overall acceptability was conducted using a nine-point hedonic scale. Proximate analyses assessed moisture, ash, fiber, protein, fat, carbohydrates, energy, calcium, and iron content. Statistical analysis was performed using the Student’s t-test.

Results: Sensory evaluation revealed that products with 15% *A. racemosus* powder (T3) scored highest for all attributes. Nutritional analysis indicated significant increases in fiber (3.12 ± 0.47 g vs. 1.74 ± 0.03 g), carbohydrates (76.07 ± 0.33 g vs. 64.72 ± 0.77 g), calcium (95.26 ± 0.62 mg vs. 14.5 ± 0.48 mg), and energy (349 ± 0.64 kcal vs. 322.2 ± 0.38 kcal) in T3 compared to the control (T0).

Conclusion: Incorporating *A. racemosus* root powder into noodles and pasta enhances sensory appeal and nutritional value, offering a functional food option rich in fiber, calcium, and energy. This approach addresses consumer demand for health-promoting and natural ingredients in processed foods. Further research on long-term health benefits is recommended.

## Introduction

The growing global emphasis on functional foods has driven innovations aimed at integrating health-promoting ingredients into everyday diets. Functional foods are designed not only to provide basic nutrition but also to offer additional benefits that contribute to disease prevention and overall health improvement [1]. Among these, medicinal plants have emerged as a key resource for developing nutritionally enriched products, owing to their bioactive components and diverse therapeutic properties. One such plant, *Asparagus racemosus *(*A. racemosus*) Willd., commonly known as Shatavari, has garnered significant attention for its multifaceted medicinal potential and rich nutritional profile [2].

*A. racemosus* belongs to the *Asparagaceae* family and has been extensively utilized in traditional medicine systems, including Ayurveda, Unani, and Siddha [3]. The plant exhibits a remarkable array of medicinal properties, including aphrodisiac, antioxidant, immunostimulant, antihepatotoxic, antibacterial, antidiabetic, anticarcinogenic, antidiarrheal, antiulcerogenic, and antioxytocic effects [4]. These attributes are primarily attributed to its bioactive compounds, such as steroidal saponins, flavonoids, alkaloids, and essential oils [2]. Comprehensive analyses of *A. racemosus* cultivated in India have highlighted its rich nutritional composition, with a total ash content of 6.2 ± 0.1%, water-soluble ash of 1.9 ± 0.0%, and acid-insoluble ash of 1.7 ± 0.0% [5]. Moreover, the plant's roots contain significant levels of phenolic compounds (14.0 ± 0.1 mg gallic acid equivalents per gram of extract), flavonoids (7.1 ± 0.2 mg quercetin equivalents per gram of extract), and saponins (4.5 ± 0.2%), which collectively contribute to its antioxidant and therapeutic efficacy [6].

The absence of harmful microorganisms, such as *Staphylococcus aureus*, *Escherichia coli*, coliforms, *Salmonella*, yeast, and molds, coupled with the lack of heavy metals like mercury, arsenic, cadmium, and lead, underscores the safety of *A. racemosus* for human consumption. The energy value of *A. racemosus* roots has been reported to be approximately 22 kcal/100 g, further enhancing its suitability as a dietary component [5]. Additionally, its roots are a rich source of trace minerals, including zinc (53.15 mg/g), cobalt (22.00 mg/g), manganese (19.98 mg/g), and copper (5.29 mg/g), alongside essential nutrients such as selenium, potassium, magnesium, and calcium [7]. These attributes position *A. racemosus* as a promising ingredient for functional food development.

Extrusion cooking, a widely employed technique in the food industry, offers a versatile platform for incorporating nutritionally rich and bioactive ingredients like *A. racemosus* into innovative food products. This high-temperature, short-time processing method is utilized for manufacturing a variety of products, including snack foods, breakfast cereals, noodles, and pasta. Extrusion not only enhances the sensory appeal of food but also preserves or enhances its nutritional properties by improving the digestibility of starch and protein. The global market for ready-to-eat and snack foods is expanding, with consumers increasingly seeking products that combine convenience with health benefits. However, traditional extruded products, often derived from cereal flour, are low in protein and essential amino acids, resulting in limited biological value. To address this, the incorporation of nutrient-dense and functional ingredients, such as *A. racemosus*, into extruded foods is gaining traction.

Incorporating *A. racemosus* into extruded snack foods offers an opportunity to create products that are not only nutritionally enriched but also possess therapeutic benefits. The plant’s rich profile of secondary metabolites, including phenolics, saponins, flavonoids, and alkaloids, can impart antioxidant and health-promoting properties to extruded products. Moreover, the incorporation of *A. racemosus* aligns with the growing consumer demand for plant-based, natural, and functional ingredients in processed foods. By leveraging the nutritional and therapeutic potential of *A. racemosus*, it is possible to address nutritional deficiencies while catering to the evolving preferences of health-conscious consumers.

The fortification of whole wheat noodles and whole wheat-semolina pasta with *A. racemosus* was undertaken to explore its potential as a functional ingredient for creating nutrient-rich, health-beneficial food products.

## Materials and methods

Procurement of A. racemosus roots and preparation of fine powder

The plant was procured from farms in Nepalapur, Sitapur, Uttar Pradesh, India. Mature roots of *A. racemosus* were uprooted. Authentic roots of *A. racemosus* were thoroughly washed with tap water. The roots were then cut into small pieces and dried under shade for three months. Once fully dried, the roots were ground into a coarse powder using a pestle and mortar. This coarse powder was filtered through muslin cloth to obtain a fine powder, which was subsequently stored in an airtight screw-capped glass bottle.

Preparation of wholewheat noodles

The formulation of the dough for whole wheat noodles involved varying proportions of whole wheat flour (ranging from 100 g to 70 g) and crude powder derived from *A. racemosus* roots (ranging from 0 g to 30 g) to achieve a total weight of 100 g. *A. racemosus* roots are bland in taste, thus its varying quantity is added to prepare a best acceptable product. A total of 100 g of product is prepared in which the base ingredient (wheat flour) is replaced with varying quantities of *A. racemosus* root powder. Hence there is a increase in the percentage of *A. racemosus* root powder and a decrease in the percentage of base ingredient in both the products prepared. Four distinct dough samples were prepared and categorized as follows: (1) Sample A (T0): 100 g whole wheat flour and 0 g *A. racemosus* root powder; (2) Sample B (T1): 95 g whole wheat flour and 5 g *A. racemosus* root powder; (3) Sample C (T3): 90 g whole wheat flour and 10 g *A. racemosus* root powder; and (4) Sample D (T4): 85 g whole wheat flour and 15 g *A. racemosus* root powder.

**Figure 1:**
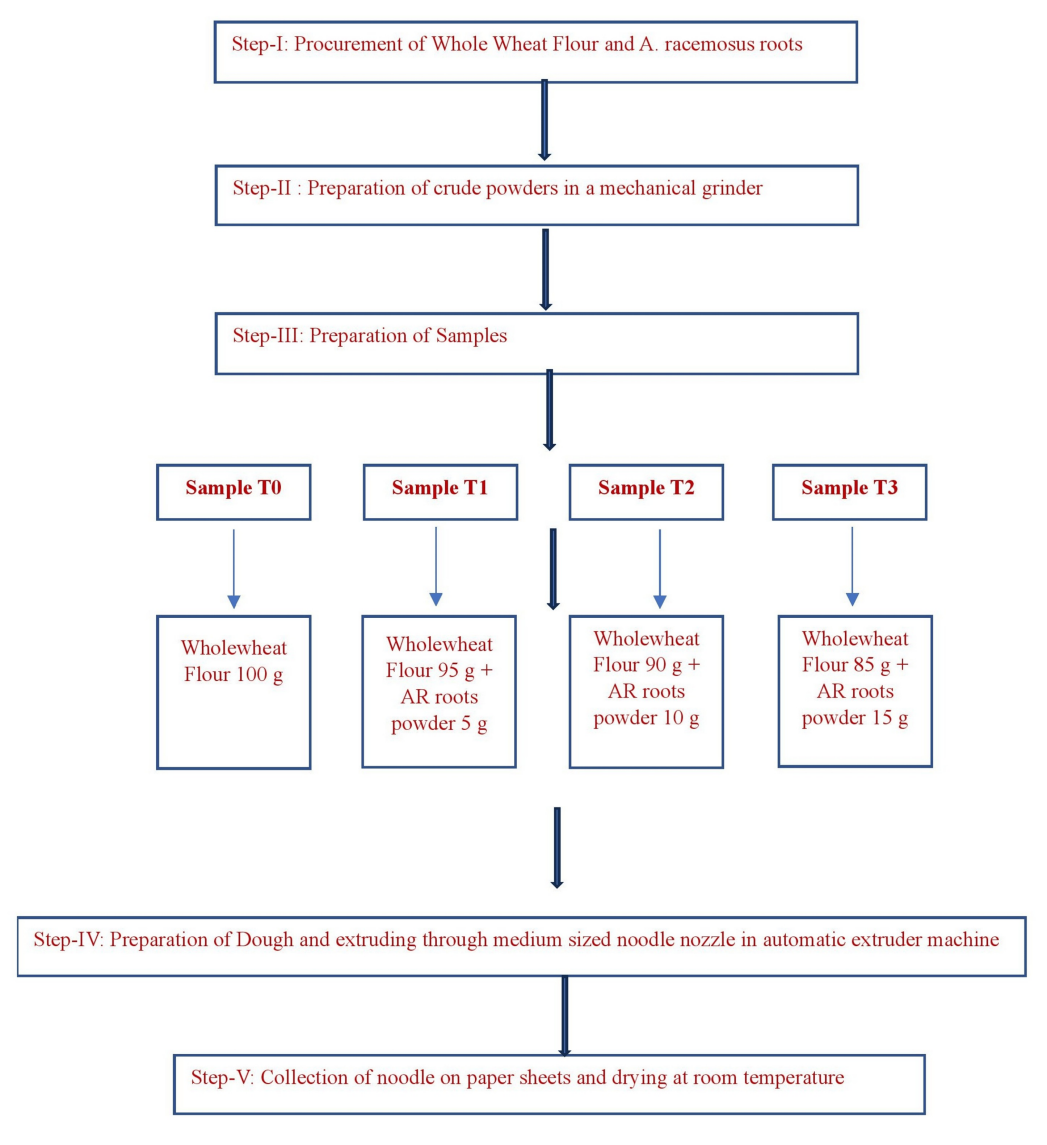
Steps in preparation of whole wheat noodles incorporated with A. racemosus root powder. AR: *Asparagus (A.) racemosus*.

Each set of ingredients was introduced into a Kent Pasta Maker Automatic Machine (Adishwar India Limited, Bengaluru, India), equipped with a medium pore-sized noodle extrusion nozzle. The ingredients were subjected to high pressure and temperature, resulting in a well-kneaded, dense dough. The extruded noodles were collected on sheets and subsequently allowed to air dry at room temperature. Figure 1 describes the steps involved in the preparation of whole wheat noodles incorporated with *A. racemosus *root powder.

Preparation of wholewheat and semolina mixed pasta

**Figure 2:**
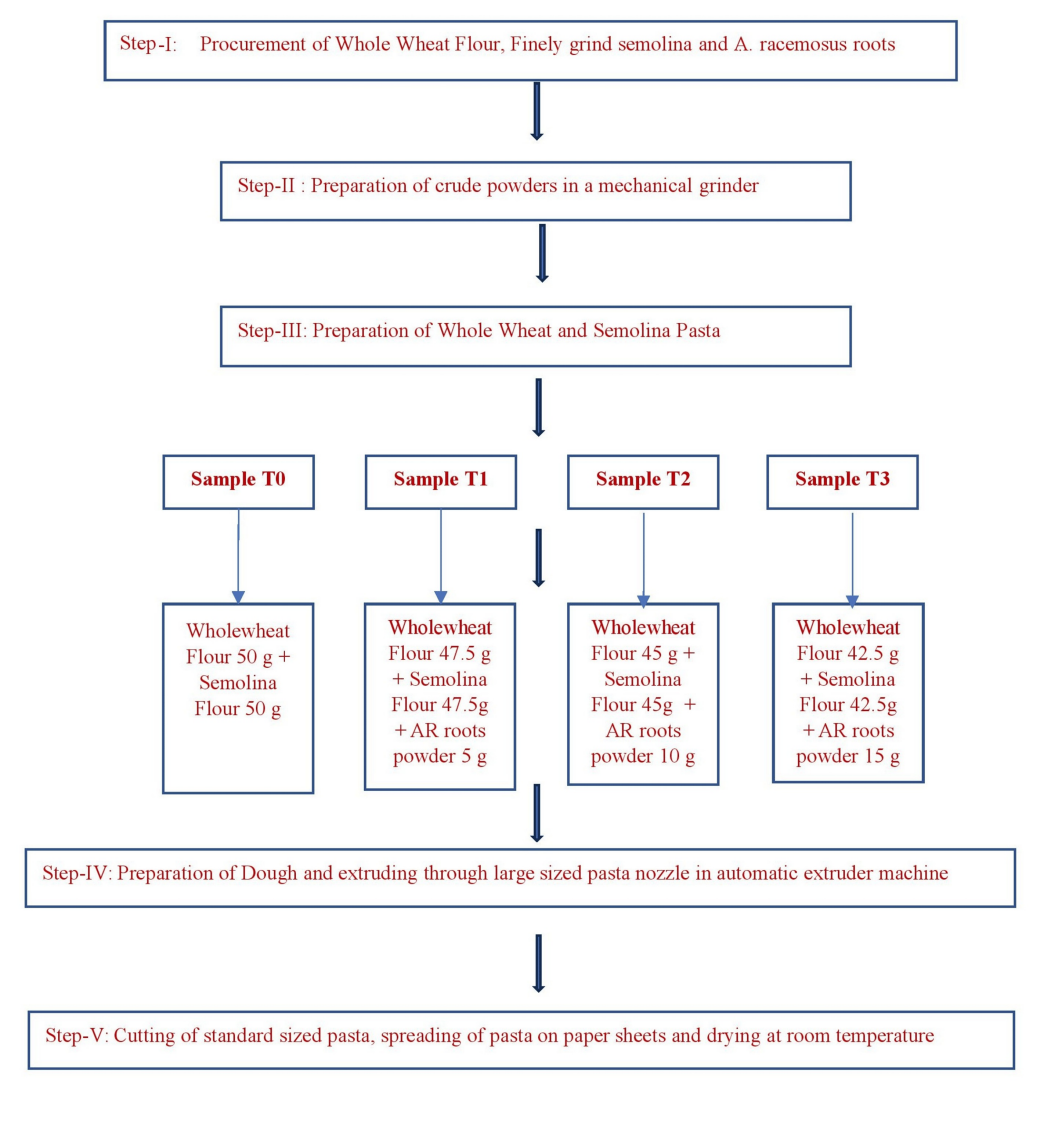
Steps in preparation of whole wheat and semolina pasta incorporated with A. racemosus root powder. AR: *Asparagus (A.) racemosus*.

The dough formulations for wholewheat and semolina pasta were composed of varying proportions of wholewheat flour (ranging from 50 g to 42.5 g), finely ground semolina (ranging from 50 g to 42.5 g), and a crude powder of *A. racemosus* roots (ranging from 0 g to 15 g) to achieve a total weight of 100 g. Four distinct dough samples were prepared as follows: (1) Sample 1 (T0) contained 0 g of *A. racemosus* roots, 50 g of wholewheat flour, and 50 g of semolina; (2) Sample 2 (T1) contained 5 g of *A. racemosus* roots, 47.5 g of wholewheat flour, and 47.5 g of semolina; (3) Sample 3 (T3) contained 10 g of *A. racemosus* roots, 45 g of wholewheat flour, and 45 g of semolina; and (4) Sample 4 (T4) contained 15 g of *A. racemosus* roots, 42.5 g of wholewheat flour, and 42.5 g of semolina. Each mixture was processed using a Kent Pasta Maker Automatic Machine (Adishwar India Limited, Bengaluru, India) equipped with a large-pore pasta extrusion nozzle. The ingredients were kneaded under high pressure and temperature to form a dense dough, which was then extruded into uniform pasta shapes, collected on sheets, and allowed to dry at room temperature. Figure 2 describes the steps involved in the preparation of whole wheat and semolina pasta incorporated with *A. racemosus* root powder.

Sensory evaluation of A. racemosus roots added noodles and pasta

The noodles and pasta samples were evaluated for color, texture, taste, and overall acceptability using a nine-point hedonic scale (ranging from 0 to 9) [8]. A panel of 15 experts conducted the assessment. The scores assigned by the experts for each parameter were averaged, and the overall acceptability of the products was determined based on these averaged scores.

Proximate analysis of wholewheat noodles and wholewheat and semolina pasta

Moisture Content

Five grams of fine powder from each sample (noodles and pasta) were separately placed in pre-weighed porcelain dishes. The dishes with samples were then re-weighed and subsequently placed in a 130°C oven for two hours. Following this, the dishes were transferred to a desiccator at room temperature. The dishes were weighed at 30-minute intervals until a constant weight was achieved. Finally, the difference in the % moisture content of the sample was calculated by the formula:

% Moisture content = 100 x (W1-W2) / W1-W

Where W1 = Weight in grams of the dish with the material before drying; W2 = Weight in grams of the dish with the material after drying to constant weight; and W = Weight in grams of the empty dish.

Total Ash

The total ash content in noodle and pasta samples was determined according to the Association of Official Analytical Chemists (AOAC) (2023) method [9]. A 5.0 g sample was placed into a pre-weighed clean crucible. The crucible, with its lid partially open, was initially heated over a low Bunsen burner until no more fumes were emitted. Subsequently, the crucible without the lid was transferred to a furnace and heated overnight (10 hours) at 550°C. After heating, the lid was replaced to prevent the loss of any volatile ash. The crucible was then allowed to cool in a desiccator, and its weight was recorded once the sample had turned grey. The % ash percent in the sample was calculated as follows:

Total ash = W1-W2*100 / Ws

Where W1 = Weight of the crucible with sample; W2 = Weight of the crucible with ash; and Ws = Weight of the sample

Crude Fiber

The AOAC (2023) test method was used for the estimation of crude fiber [9]. Crude powder of 2.0 grams from the sample (noodles and pasta) was placed in a 500 mL round-bottom flask. A measured volume of 200 mL of dilute sulfuric acid was added to the flask. The mixture was heated on a hot plate and maintained at boiling for 30 minutes. Following the heating period, the mixture was filtered using a muslin cloth, and the residue was thoroughly washed with water.

A 200 mL aliquot of 0.313 N sodium hydroxide (NaOH) solution was transferred into a 500 mL round-bottom flask, to which the acid-digested sample was added. The mixture was then boiled for 30 minutes, allowed to cool, and subsequently filtered through muslin cloth. The residue was washed repeatedly with water. The residue was placed in a pre-weighed crucible (designated as W1) and dried in a hot air oven at 130°C for two hours, followed by cooling in a desiccator. The crucible was then subjected to a muffle furnace at 550°C for two hours. After cooling, the crucible was weighed again (designated as W2). The total fiber content in the sample was determined by calculating the difference between W2 and W1. The percentage fiber content in the sample was then calculated using the following formula:

Crude fiber = W1-W2 x 100 / Ws

Where W1 = Weight of the crucible with sample; W2 = Weight of the crucible with ash; and Ws = Weight of the sample.

Total Fat Content

A bottle and its lid were placed in an incubator set at 105°C overnight and weighed periodically until a constant weight was achieved. Approximately 3-5 g of crude powder from the sample (noodles and pasta) were weighed on filter paper, transferred to an extraction thimble, and then inserted into a Soxhlet extractor. The Soxhlet apparatus was connected to a bottle containing 200 mL of petroleum ether, which was placed on a heating mantle. Water was circulated through the condenser to maintain cooling, while the heating mantle was activated. Heating was maintained at a rate of 150 drops per minute for a duration of 14 hours. After extraction, the solvent was removed using a vacuum condenser. The bottle was then incubated at 80-90°C until the solvent evaporated completely and the bottle was dry. The dry bottle, with a partially covered lid, was transferred to a desiccator to cool. The bottle was reweighed to determine the weight of the dried content. The percentage of fat present in the sample was then calculated using the following formula:

Crude fat % = W2 - W1*100 / Ws

Where W2 = Weight of the flask with fat; W1 = Weight of the flask; and Ws = Weight of the sample

Protein

The protein content in the samples (noodles and pasta) was determined using the Kjeldahl method. This procedure involved measuring the nitrogen content and then converting it to protein content by applying a protein factor of 6.25. A quantity of 0.5 to 1.0 grams of the crude powder sample was placed in a digestion flask, to which 5 g of Kjeldahl catalyst (comprising potassium sulfate {K₂SO₄} and copper sulfate {CuSO₄}) and 200 milliliters of concentrated sulfuric acid were added. A control flask, containing no sample, was prepared under the same conditions. Both flasks were positioned at an incline and heated gently until the frothing ceased and the solution was clear. Subsequently, 60 milliliters of distilled water were added. The flasks were then connected to a digestion bulb with a condenser, ensuring the tip of the condenser was immersed in standard acid, and five to seven drops of the indicator were placed in the receiver. The mixture was thoroughly mixed and heated until all ammonia was distilled. After removing the receiver, the tip of the condenser was washed, and the excess standard acid was titrated with a standard alkali solution. The protein content percentage in the sample was then calculated based on these measurements.

Protein % = (A-B) x N x 14.007 x 6.25 / W

Where A = Volume (ml) of 0.2 N HCl used in sample titration; B = Volume (ml) of 0.2 N HCl used blank titration; N = Normality of hydrochloric acid (HCl); W = Weight (g) of sample; 14.007 = Atomic weight of nitrogen; and 6.25 = Protein nitrogen conversion factor.

Carbohydrates

The carbohydrate content in a 100 g sample (noodles and pasta) was determined according to the nitrogen-free method described by AOAC (2023) [9]:

% Carbohydrate (NFE) = 100 - (moisture + protein + fat + ash + crude fiber)

Calcium

A 5.0 g sample of crude powder (noodles and pasta) was placed in a 100 ml beaker, to which 8.0 ml of concentrated sulfuric acid and 10 ml of concentrated nitric acid were added. The beaker was subsequently positioned on a hot plate and carefully heated until the reaction subsided. To prevent charring, additional aliquots of concentrated nitric acid were periodically introduced. Following this, the solution was allowed to cool and then diluted with 10 ml of double-distilled water, followed by boiling until fumes were produced. Hydrogen peroxide was added each time the solution was heated to the fuming state until the residue was decolorized or no further reduction in pale yellow color was observed. The solution was then transferred to a 100 ml volumetric flask and brought to volume. A 25.0 ml aliquot of this digest was pipetted into a beaker, to which NaOH solution was added to adjust the pH to 12-13. Two drops of Solochrome Dark Blue dye were then introduced, and the mixture was immediately titrated with 0.01 M EDTA (Ethylenediaminetetraacetic acid) solution until a blue endpoint was achieved. Titration method was used to determine the calcium content.

Iron

A 25 g sample (noodles and pasta separately) was placed in a dish and treated with 20% sulfuric acid. The mixture was thoroughly agitated to ensure complete wetting of the sample. The sample was then heated in an oven at approximately 110°C. Following this, the sample was further heated on a soft flame to remove all volatile and easily combustible materials. The dish was subsequently transferred to a furnace and ashed at 500°C for six to eight hours, after which it was allowed to cool. Once the carbon-free ash was obtained, 1.0 ml of concentrated nitric acid and 10 ml of water were added to dissolve the ash. The resulting solution was transferred to a 50 ml volumetric flask. Next, 10 ml of concentrated hydrochloric acid was added to the dish and heated, and the solution was then transferred to the volumetric flask. A blank sample, prepared with the same reagents but without the sample, was also prepared. The absorbance of both the sample solution and the blank was measured using an atomic absorption spectrophotometer. The iron concentration was determined by comparing the sample's absorbance with a standard calibration curve.

Calorific Value

The calorific value (energy value) of a 100 g sample (noodles and pasta) was calculated by using the following formula:

Energy value = (% protein x 4) + (% fat x 9) + (% carbohydrate x 4).

Statistical analysis

Values of samples with and without *A. racemosus* roots were compared statistically by employing the Student’s t-test. Each value is the average value ± standard error (SE) of three independent experiments. p-value less than 0.05 was taken as significant.

## Results

Sensory evaluation results for whole wheat noodles

Table 1 displays the sensory evaluation scores for whole wheat noodle samples (T0 to T3), presented as mean ± SE from 15 independent assessors. The data indicates that sample T3 received the highest scores, with 8.50 for color, 7.64 for texture, 8.57 for taste, and 8.29 for overall acceptability.

**Table 1:** Sensory evaluation of whole wheat noodles. TO: 100 g whole wheat flour and 0 g *A. racemosus* root powder; T1: 95 g whole wheat flour and 5 g *A. racemosus* root powder; T2: 90 g whole wheat flour and 10 g *A. racemosus *root powder; T3: 85 g whole wheat flour and 15 g *A. racemosus *root powder. The sensory evaluation scores are presented as mean ± standard error (S.E.) based on assessments from 15 independent panelists. The total possible score range for each sensory attribute (color, texture, taste, and overall acceptability) is 1 to 9, with 1 representing the lowest acceptability and 9 representing the highest acceptability.

Sample	Color	Texture	Taste	Overall acceptability
T0	7.67 (±0.72)	6.54 (±0.58)	7.20 (±0.77)	7.30 (±0.62)
T1	7.87 (±0.70)	7.77 (±0.48)	7.75 (±0.59)	7.78 (±0.83)
T2	8.28 (±0.62)	7.63 (±0.24)	8.53 (±0.60)	8.20 (±0.37)
T3	8.50 (±0.22)	7.64 (±0.35)	8.57 (±0.16)	8.29 (±0.29)

Color

The mean scores for color, texture, taste, and overall acceptability of whole wheat noodles, as evaluated by 15 independent assessors (five trained, five semi-trained, and five untrained, all independent of each other; a total of 15) are presented in Table 1. The highest average sensory score for color was observed in T3 (8.50 ± 0.22), followed by T2, T1, and T0, with scores of 8.28 ± 0.62, 7.87 ± 0.70, and 7.67 ± 0.72, respectively. This indicates a significant difference in color between the control and samples containing *A. racemosus*, suggesting that the quantity of *A. racemosus* added affects the product's color. The color of the developed product becomes darker with the addition of *A. racemosus*, while its acceptability increases proportionally, demonstrating that the addition significantly influences the product's sensory attributes.

Texture

The mean texture score was highest in T3 (7.64 ± 0.35), followed by T2 (7.63 ± 0.24), T1 (7.77 ± 0.48), and T0 (6.54 ± 0.58), indicating a significant variation in texture between the control and treated food products. The incorporation of varying amounts of *A. racemosus* influenced the texture of the developed noodles, thereby enhancing their overall acceptability.

Taste Attribute

The average sensory score for the taste attribute was highest in T3 (8.57 ± 0.16), followed by T2 (8.53 ± 0.60), T1 (7.75 ± 0.59), and T0 (7.20 ± 0.77). The incorporation of *A. racemosus* root powder into the base ingredients progressively enhanced the taste as the concentration of the test ingredient increased, demonstrating a significant influence in this treatment.

Overall Acceptability

The mean score for overall acceptability was highest in T3 (8.29 ± 0.29), followed by T2 (8.20 ± 0.37), T1 (7.78 ± 0.83), and T0 (7.30 ± 0.62). This indicates a significant difference in overall acceptability between the control and the food product with added *A. racemosus*. The percentage of *A. racemosus* addition influenced the overall acceptability of the developed products.

Sensory evaluation results for whole wheat and semolina mixed pasta

Table 2 presents the sensory evaluation results for whole wheat and semolina mixed pasta samples (T0 to T3) as average ± S.E. The data indicate that sample T3 received the highest color, texture, taste, and overall acceptability scores.

**Table 2:** Sensory evaluation of whole wheat and semolina mixed pasta. T0: 50 g whole wheat flour, 50 g semolina, 0 g *A. racemosus* root powder; T1: 47.5 g whole wheat flour, 47.5 g semolina, 5 g *A. racemosus* root powder; T2: 45 g whole wheat flour, 45 g semolina, 10 g *A. racemosus* root powder; T3: 42.5 g whole wheat flour, 42.5 g semolina, 15 g *A. racemosus* root powder. The total possible score range for each sensory attribute (color, texture, taste, and overall acceptability) is 1 to 9, where 1 indicates the lowest acceptability and 9 indicates the highest acceptability.

Sample	Color	Texture	Taste	Overall acceptability
T0	5.66 (±0.19)	5.46 (±0.45)	5.83 (±0.21)	5.68 (±0.56)
T1	6.46 (±0.54)	6.52 (±0.30)	6.48 (±0.43)	6.41 (±0.69)
T2	7.25 (±0.27)	7.56 (±0.08)	7.36 (±0.27)	7.34 (±0.23)
T3	8.52 (±0.38)	7.73 (±0.36)	8.65 (±0.23)	8.17 (±0.19)

Color

The mean sensory ratings for various attributes, including color, texture, taste, and overall acceptability, of whole wheat and semolina pasta are presented in Table 2. The highest average sensory score for color was observed in T3 (8.52 ± 0.38), followed by T2, T1, and T0, with scores of 7.25 ± 0.27, 6.46 ± 0.54, and 5.66 ± 0.19, respectively. These results indicate a significant difference in color between the control and *A. racemosus*-enhanced products, suggesting that the percentage addition of crude *A. racemosus *root powder affects the product's color. Specifically, the color of the developed product darkens as the amount of *A. racemosus *crude powder increases, while its acceptability also improves.

Texture

The mean texture score was highest in T3, recorded at 7.73 ± 0.36, followed by T2 at 7.56 ± 0.08, T1 at 6.52 ± 0.30, and T0 at 5.46 ± 0.45. This indicates a significant difference in texture between the control and the food products supplemented with *A. racemosus*. The incorporation of varying amounts of *A. racemosus* influenced the texture of the developed pasta, enhancing the acceptability of the fortified product.

Taste

The mean sensory evaluation for the taste attribute was highest in sample T3 (8.65 ± 0.23), followed by T2, T1, and T0, which scored 7.36 ± 0.27, 6.48 ± 0.43, and 5.83 ± 0.21, respectively. The incorporation of *A. racemosus* root powder into the base ingredients resulted in a progressive enhancement of taste, correlating with increased concentrations of the test ingredient.

Overall Acceptability

The overall acceptability score was highest in T3 (8.17±0.19), followed by T2 (7.34±0.23), T1 (6.41±0.69), and T0 (5.68±0.56). This indicates a significant difference in overall acceptability between the control and the food products fortified with *A. racemosus*. The incorporation of *A. racemosus* powder significantly influences the acceptability of the fortified products, with higher quantities of the powder correlating with greater acceptability.

Nutritional composition of A. racemosus roots added whole wheat noodles compared to those without

Table 3 presents a comparison of the nutritional composition of whole wheat noodles fortified with *A. racemosus* root powder (T3) and those without (T0). The moisture content was higher in T3 (12.42±0.13) compared to T0 (11.02±0.21). The calculated t-value for the difference in moisture content between T0 and T3 was 5.66, indicating a significant difference. Additionally, increasing the amount of *A. racemosus* root powder was associated with an increase in moisture content. The ash content was also higher in T3 (1.69±0.06) compared to T0 (1.52±0.12), but the t-value for this difference was 1.26, suggesting an insignificant difference.

**Table 3:** Nutritional composition of whole wheat noodles. T0: 100 g whole wheat flour, 0 g *A. racemosus* root powder; T3: 85 g whole wheat flour, 15 g *A. racemosus* root powder. p-value less than 0.05 was taken as significant.

S. No.		Sample		Difference (T0 -T3=D)	Student's t-value (Calculated)	Standard error of difference	p-value
		T_0_	T_3_				
1.	Moisture (g/100g)	11.02 (±0.21)	12.42 (±0.13)	-1.40	5.66	0.24	0.05
2.	Ash (g/100g)	1.52 (±0.12)	1.69 (±0.06)	-0.17	1.26	0.13	0.10
3.	Fiber (g/100g)	1.74 (±0.03)	3.12 (±0.47)	-1.38	2.93	0.47	0.034
4.	Fat (g/100g)	1.56 (±0.37)	1.18 (±0.11)	0.38	0.98	0.38	0.23
5.	Protein (g/100g)	12.32 (±0.53)	12.64 (±0.29)	- 0.32	0.52	0.60	0.10
6.	Carbohydrates (g/100g)	64.72 (±0.77)	76.07 (±0.33)	-11.35	13.54	0.83	0.02
7.	Energy (Kcal/100g)	322.2 (±0.38)	349 (±0.64)	-26.80	36.00	0.74	0.05
8.	Calcium (mg/100g)	14.5 (±0.48)	95.26 (±0.62)	-80.76	102.99	0.78	0.05
9.	Iron (mg/100g)	0.92 (±0.59)	1.61 (±0.54)	-0.69	.86	0.80	0.73

The incorporation of *A. racemosus* root powder into products demonstrated a percentage increase in ash content, though this difference was not statistically significant. The fiber content in the T3 formulation (3.12±0.47) was significantly higher compared to the control T0 (1.74±0.03), with a calculated T-value of 2.93 indicating statistical significance. Conversely, an increase in *A. racemosus* root powder led to a decrease in fat content, with T0 showing higher fat content (1.56±0.37) than T3 (1.18±0.11), though this change was not statistically significant.

The protein content in whole wheat noodles enriched with *A. racemosus* root powder also increased. T3 had a higher protein content (12.64±0.29) compared to T0 (12.32±0.16), but the T-value of 0.52 indicated no statistical significance. The carbohydrate content was significantly higher in T3 (76.07±0.33) compared to T0 (64.72±0.77), with a T-value of 13.54. Additionally, the energy content was significantly higher in T3 (349±0.64) compared to T0 (322.20±0.38), with a highly significant T-value of 36.

Calcium content was markedly higher in T3 (95.26±0.62) versus T0 (14.50±0.48), with a T-value of 102.99 indicating a highly significant difference. Iron content also increased with the addition of *A. racemosus* root powder, with T3 having higher iron content (1.61±0.54) compared to T0 (0.92±0.59), although this difference was statistically insignificant with a T-value of 0.86.

Nutritional composition of A. racemosus roots added whole wheat and semolina pasta compared to control

Table 4 shows the nutritional composition of whole wheat and semolina pasta enriched with *A. racemosus* root powder (T3) compared to the control (T0). Moisture content was higher in T3 (11.35±0.12) compared to T0 (10.50±0.43), with an insignificant T-value of 1.90. The ash content in T3 (5.13±0.24) was significantly higher than in T0 (1.35±0.05), with a T-value of 15.41. The fiber content was also higher in T3 (3.82±0.37) compared to T0.

**Table 4:** Nutritional composition of whole wheat and semolina mixed pasta. T0: 50 g whole wheat flour, 50 g semolina, 0 g *A. racemosus* root powder; T3: 42.5 g whole wheat flour, 42.5 g semolina, 15 g *A. racemosus* root powder. p-value less than 0.05 was taken as significant.

S. No.		Sample		Difference (T0 -T3=D)	Student's t-value (calculated)	Standard error of difference	p-value
		T_0_	T_3_				
1.	Moisture (g/100g)	10.5 (±0.43)	11.35 (±0.12)	-0.85	1.90	0.44	0.62
2.	Ash (g/100g)	1.35 (±0.05)	5.13 (±0.24)	-3.78	15.41	0.24	0.05
3.	Fiber (g/100g)	1.62 (±0.28)	3.82 (±0.37)	-2.20	4.74	0.46	0.05
4.	Fat (g/100g)	2.90 (±0.30)	2.23 (±0.49)	0.67	1.16	0.57	0.17
5.	Protein (g/100g)	8.74 (±0.67)	9.26 (±0.21)	-0.52	0.74	0.70	0.67
6.	Carbohydrates (g/100g)	63.3 (±0.56)	70.03 (±0.38)	-6.7	9.9	0.67	0.031
7.	Energy (Kcal/100g)	314.3 (±0.43)	355.23 (±0.67)	-40.89	51.36	0.79	0.027
8.	Calcium (mg/100g)	12.6 (±0.66)	150.2 (±0.47)	-137.58	169.80	0.81	0.019
9.	Iron (mg/100g)	1.54 (±0.08)	1.76 (±0.09)	-0.22	1.82	0.12	0.35

Overall, the addition of *A. racemosus* root powder generally increased the nutritional quality of the finished products, particularly in terms of fiber, protein, carbohydrate, energy, and calcium content, with varying degrees of statistical significance.

The statistical analysis of the fiber content between the control product (T0) and the widely accepted product (T3) yielded a t-value of 4.74, indicating a statistically significant difference. An increase in the percentage of *A. racemosus* root powder in the final products corresponded to an increase in fiber content. However, the fat content was greater in T0 (2.90±0.30) compared to T3 (2.23±0.49). The t-value for the difference in fat content between T0 and T3 was 1.16, which is not statistically significant.

The protein content in whole wheat and semolina pasta enriched with *A. racemosus* root powder showed an increase in T3 (9.26±0.21) compared to T0 (8.74±0.67). The t-value for the protein content difference between T0 and T3 was 0.74, suggesting an insignificant difference statistically.

The carbohydrate content was higher in T3 (70.03±0.38) than in T0 (63.32±0.56). The t-value for the carbohydrate content difference between T0 and T3 was 9.9, indicating a statistically significant difference.

The energy content was also higher in T3 (355.23±0.67) compared to T0 (314.34±0.43). The t-value for the difference in energy content between T0 and T3 was 51.36, reflecting a highly significant increase.

Calcium content was notably higher in T3 (150.2±0.47) compared to T0 (12.62±0.66). The t-value for the difference in calcium content between T0 and T3 was 169.80, indicating a highly significant difference.

Iron content in whole wheat and semolina pasta with added *A. racemosus* root powder increased with higher percentages of the powder. T3 (1.76±0.09) had a higher iron content compared to T0 (1.54±0.08). The t-value for the difference in iron content between T0 and T3 was 1.82, suggesting an insignificant difference statistically.

## Discussion

The incorporation of *A. racemosus* root powder into whole wheat noodles and semolina-based pasta significantly improved sensory attributes and nutritional quality. These findings align with prior research on the functional benefits of bioactive compounds in fortifying food products.

Sensory attributes

The sensory evaluation results consistently showed that T3 samples, containing the highest concentration of *A. racemosus* root powder, were rated superior across attributes such as color, texture, taste, and overall acceptability. A previous study reported that the addition of natural bioactive ingredients, such as herbal powders, enhances sensory properties due to their characteristic color, flavor, and textural contributions [10]. The darkening of color observed with higher *A. racemosus* concentrations is consistent with a report by Domínguez et al., who noted similar changes in products enriched with herbal ingredients, attributing this to the natural pigments in plant-based additives [11].

Furthermore, improvements in texture, taste, and overall acceptability with increasing *A. racemosus* content corroborate findings, where herbal fortification improved the palatability and mouthfeel of food products [12].

Nutritional quality

The enhancement in the nutritional profile of fortified products aligns with the functional properties of *A. racemosus*. The significant increase in fiber content in T3 formulations (3.12 g/100g for noodles and 3.82 g/100g for pasta) is consistent with previous studies indicating that plant-based additives contribute to dietary fiber enrichment [13]. Fiber's role in promoting gut health and aiding digestion underscores the potential health benefits of these fortified products [14].

Similarly, the marked increase in calcium content (95.26 mg/100g in noodles and 150.2 mg/100g in pasta) is significant. This observation resonates with prior work which highlighted the calcium-rich nature of certain herbal ingredients [15]. The potential for *A. racemosus* root powder to serve as a calcium enhancer suggests its utility in addressing micronutrient deficiencies, particularly in populations prone to osteoporosis [16].

The significant increase in carbohydrate and energy content in T3 samples is attributable to the compositional changes introduced by the addition of *A. racemosus*. These findings mirror those who observed a similar increase in energy density when fortifying wheat-based products with functional ingredients [17].

While the study demonstrated notable improvements in protein and iron content with *A. racemosus* incorporation, the changes were not statistically significant. These findings are consistent with prior reports indicating that while herbal fortification can enhance micronutrient content, the degree of improvement depends on the concentration and bioavailability of the fortifications used [18].

The reduction in fat content in T3 samples (1.18 g/100g for noodles and 2.23 g/100g for pasta) compared to controls is noteworthy, suggesting the potential of *A. racemosus* as a low-fat fortification option. Similar trends were reported by studies where herbal fortification resulted in a dilution effect on fat content due to the high fiber and moisture content of the additives [19,20].

Limitations

Despite promising results, the study had some limitations. The sample size for sensory evaluation was limited, which may affect the generalizability of findings. Moreover, while nutritional enhancements were evident, the bioavailability of added nutrients was not evaluated. Further research is required to assess the long-term acceptability, stability, and health benefits of fortified products, as well as to explore the impact of *A. racemosus* root powder on larger populations and diverse dietary contexts.

Implications and future directions

The study underscores the dual benefits of *A. racemosus* fortification in enhancing sensory appeal and nutritional quality, making it a promising ingredient for developing functional foods. The significant increases in calcium and fiber content highlight the potential of these products to address nutrient gaps in vulnerable populations. However, further research is needed to explore bioavailability and long-term health benefits associated with consuming *A. racemosus*-fortified foods. Future studies should also investigate consumer acceptance in larger demographic cohorts and assess the shelf stability of fortified products under various storage conditions. Additionally, exploring the fortification potential of *A. racemosus* in other staple foods could expand its application in addressing nutritional deficiencies globally.

## Conclusions

The study demonstrated that the incorporation of *A. racemosus* root powder into whole wheat noodles and whole wheat-semolina pasta significantly improved their sensory attributes, including color, texture, taste, and overall acceptability. Additionally, the fortified products exhibited enhanced nutritional profiles, particularly in terms of fiber, carbohydrate, energy, and calcium content, with varying degrees of statistical significance. The highest acceptability was observed in the T3 formulation, which contained the highest concentration of *A. racemosus* root powder. These findings highlight the potential of *A. racemosus* as a functional ingredient for developing nutrient-rich and sensory-appealing food products.
